# Editorial: Recent advances in pharmaceutical analysis: applications and new challenges for the quality of medicines

**DOI:** 10.3389/fchem.2025.1699047

**Published:** 2025-09-19

**Authors:** Anna Borioni, Maria Elisa Crestoni, Birgit Hakkarainen, Constantinos K. Zacharis, Federica Aureli

**Affiliations:** 1 Chemical Medicines Unit, National Centre for the Control and Evaluation of Medicines, Istituto Superiore di Sanità, Rome, Italy; 2 Department of Chemistry and Technology of Drugs, Sapienza University of Rome, Rome, Italy; 3 Official Medicines Control Laboratory, Swedish Medical Products Agency, Uppsala, Sweden; 4 Laboratory of Pharmaceutical Analysis, Department of Pharmacy, Aristotle University of Thessaloniki, Thessaloniki, Greece

**Keywords:** quality of medicines, multivariate analyis, illegal medicines, pharmaceutical analysis, spectroscopy, impurities, analytical methods, biotherapeutic

This Research Topic is concerned with the exploration of the most recent advancements and developments in the field of pharmaceutical analysis, focusing on non-destructive approaches, predictive models and the application of techniques which are less frequently used in pharmaceutical analysis. The samples belonged to the legal pharmaceutical market as well as to the illegal market, being the last increasingly deceptive and difficult to spoil.

Alamri et al. applied an ultra-performance liquid chromatography-photodiode array detector (UPLC-PDA) method and a liquid chromatography tandem mass spectrometry (LC-MS/MS) method to analyse 40 samples of suspicious pregabalin samples seized from the Saudi Arabia market. The results demonstrate significant variability in pregabalin content and the presence of toxic adulterants in about 30% of the samples confirming inadequacies in illicit drug production and circulation.

Still in the field of illegal medicines, Blazewicz et al. analysed 601 samples seized from the illegal market by liquid chromatography coupled with high-resolution hybrid mass spectrometry and X-ray powder diffraction. The samples, which were reported in bodybuilding forums, were suspected to contain illegal substances such as selective estrogenic receptor modulators (SERMs), aromatase inhibitors (AIs), and preparations containing human chorionic gonadotropin (hCG). In about 65% of the samples, the declared active pharmaceutical ingredients were present, whereas in 35%, they were not. Furthermore, 6.4% of the samples in which the declared API was found, contained an additional undeclared API.

Mocarska et al. reported the applications of Attenuated total reflectance Fourier transform infrared spectroscopy and X-ray powder diffraction for analysing suspicious illegal products that have been seized by the authorities. These advantageous techniques provide consistent, reproducible results with no environmental impact, as minimal sample preparation is required and no chemical reagents or solvents are used.

Aureli et al. described a combined approach based on Liquid Chromatography-Quadrupole Time-of-Flight Mass Spectrometry/Nuclear Magnetic Resonance spectroscopy LC-MS Q-TOF/1H-NMR to identify and quantify L-dopa in dietary supplements purchased online. The study evidenced the presence on the online market of unauthorised botanical extracts containing L-dopa and amount either as stated but also overdosed or under-dosed. Given the observed increasing public interest in levodopa discussed by the authors, these findings highlight the need to control this market.

In the above-mentioned papers multimodal analytical strategies were efficiently implemented to successfully pursue high discrimination power and method robustness. All confirmed that the declared active substances in falsified and unauthorised products may be absent, substituted, adulterated and misdosed, posing a serious threat to consumer health. There is an urgent need to inform consumers and physicians about the risks of purchasing such products, frequently available through online sources.

The importance of understanding plant-based product composition was addressed in different studies. Zhang et al. proposed a new integrated strategy based on E-nose, E-tongue, LC-HR-Q-TOF-MS/MS, and electrochemical fingerprint spectra to systematically differentiate Asarum heterotropoides (AH) from Cynanchum paniculatum (CP) a medicinal plant used in Traditional Chinese Medicine the first containing toxic ingredients. This novel synergic approach allowed identification of 25 odour components in AH and 12 odour components in CP, differentiation in bitterness and astringency between the two plants, identification of about 90 components in each plant plus further distinctions based on the electrochemical fingerprint spectra. Through the combination of electrochemical fingerprint spectra with principal component analysis (PCA) or orthogonal partial least squares-discriminant analysis (OPLS-DA), the accuracy of this method reached 100%.

Hakeem et al. focused their paper on the quantification, by LC-MS/MS, of phytohormones in *Aloe vera,* a succulent plant renowned for its diverse therapeutic effects, including anti-inflammatory and antimicrobial properties. While traditional methods for detecting phytohormones in plant extracts often fall short in terms of sensitivity, selectivity, and precision, the method here described was demonstrated suitable to quantify six phytohormones in *A. vera* samples randomly selected from diverse sources with LOQ as low as 0.04 ng/mL.

Fungi also hold a significant position in the healthcare and nutrition market. *Ganoderma lucidum* evidenced antibacterial, tumour-inhibiting, and anti-inflammatory effects. Ran et al. provided a non-destructive approach to assessing *G. lucidum* quality with a focus on the ergosterol and polysaccharide content present in the different parts of the fungus. Three machine learning models—a feedforward neural network, an extreme learning machine, and a decision tree—were tested and compared to the ergosterol and polysaccharide composition determined by chemical analysis. The extreme learning machine model provided a successful approach giving good predictions for polysaccharides and ergosterol content in *G. lucidum.*

(S)-Nicotine is a chiral alkaloid extracted from Tobacco plants which is used as addictive ingredient in smoking products. In the last few decades, synthetic (S)-nicotine has gained interest due to the straightforward manufacturing process. Nisathar et al. estimated the impurity profile of thirteen different lots of synthetic nicotine in comparison with fourteen lots of nicotine extracted from plants using an in-house reversed-phase HPLC analytical method. Similar quantities of total impurities in both synthetic and tobacco-extracted nicotine were detected though synthetic nicotine lacks some impurities such as cotinine, nornicotine, nicotine-N-oxide. Both kinds of nicotine have similar high enantiomeric purity. Nitrosamines were not detected in any synthetic or extracted nicotine lots.

Another valuable plant-derived active ingredient is phytostimulin which is extracted from wheat (*Triticum vulgare*). The process is a water-based extraction procedure with not harmful solvents but in line with the principle of reduction-reuse-recycling, Ciriaco et al. performed a chemical characterization, by UHPLC-Q-Orbitrap HRMS, of the bioactive compounds in both liquid and solid wastes from the processing of wheat and evaluated their safety and efficacy profile in human cellular models. The solid waste contained the highest phenol component represented by ferulic acid at approximately 90 mg/kg and resulted a potentially starting resource in pharmaceutical and other areas such as cosmeceuticals or food.

The following two papers focus on the analysis of anti-cancer drugs selpercatinib and ponatinib. Both are protein tyrosine kinase inhibitors with complex chemical structure and an impurity profile affecting safety and effectiveness.

Xiang et al. developed and validated a High-Performance Liquid Chromatography (HPLC) method for identifying selpercatinib and its related impurities. The experimental results and the scientific rationale underpinning the process of method development were reported in a step-by-step manner. Based on the results of stress tests the method was also validated for its capacity to detect the degradation impurity that may form under storage, and transportation of selpercatinib.

Wang et al. developed and successfully validated a robust and sensitive reverse phase HPLC method to detect the process impurity and degradation product of ponatinib. In the process a novel impurity forming under oxidative degradation was detected and its chemical structure was elucidated by HR-MS and NMR. The process underlying the method development was accurately described. The limits of quantification were found in the ng/mL range.

Rivaroxaban based medicines have anticoagulant effect and are commonly used to prevent thrombosis or inhibit the enlargement of existing blood clots. A robust, precise, and selective reversed-phase high-performance liquid chromatography (HPLC) method was developed and extensively validated by Mestareehi for analyzing Rivaroxaban in tablets. The method was demonstrated to be both stability-indicating and highly sensitive, with a limit of quantification of 1 ppm.

If patients could take a lower dosage of drugs and still achieve the same therapeutic effectiveness, the harmful effects of impurities would be reduced. To achieve this, the solubility of the active pharmaceutical ingredients in an aqueous medium at a physiological pH could be increased. Production of nanomedicines is a possible approach and processing drugs with supercritical carbon is one of the methods for creating nanomedicines. In this view Hagbani et al. focused on the application of machine learning to model the solubility of ketoprofen in supercritical carbon dioxide by three regression techniques. A comprehensive visual representations and statistical examinations of the connections between temperature, pressure, and solubility was also provided. The results obtained in this study showed high performance of the machine learning model in prediction of drug solubility in supercritical CO_2._

Biological medicinal products are recognised as more complex than chemical medicines, requiring specific analytical techniques and a more holistic approach. Charge heterogeneity is a critical quality attribute of proteins and mAb-based drugs which is often analysed using separative techniques such as imaged capillary isoelectric focusing (icIEF).

Ghizzani et al. reviewed the icIEF advancements in the context of biotherapeutics drug development and their quality control. Key aspects, including sample preparation, capillary properties, carrier ampholytes, stabilizers, and detection are examined, and supported by recent literature. The wide range of variables that can influence this analytical method make it a suitable candidate for the development of Analytical Quality by Design approaches, according to ICH Q14 guideline and for applications of artificial intelligence in managing complex icIEF data sets.

In this Research Topic, we have provided the reader with examples of innovative approaches and application of predictive models. In the case of papers reporting more traditional methods, the analytical development and validation process is stepwise outlined. Our goal is that the Research Topic will be a source of inspiration for readers, encouraging the development of new lines of research and collaborative projects.

